# Volumetric capnography pre- and post-surfactant during initial resuscitation of premature infants

**DOI:** 10.1038/s41390-021-01578-4

**Published:** 2021-05-22

**Authors:** Emma E. Williams, Theodore Dassios, Katie A. Hunt, Anne Greenough

**Affiliations:** 1 Department of Woman and Children’s Health, School of Life Course Sciences, Faculty of Life Sciences and Medicine, King’s College London, London, UK; 2 Neonatal Intensive Care Centre, King’s College Hospital NHS Foundation Trust, London, UK; 3 Asthma UK Centre for Allergic Mechanisms in Asthma, King’s College London, London, UK; 4 National Institute for Health Research (NIHR) Biomedical Research Centre based at Guy’s and St Thomas’ NHS Foundation Trust and King’s College London, London, UK

## Abstract

**Background:**

Volumetric capnography allows for continuous monitoring of expired tidal volume and carbon dioxide. The slope of the alveolar plateau of the capnogram (S_III_) could provide information regarding ventilation homogeneity. We aimed to assess the feasibility of measuring S_III_ during newborn resuscitation and determine if S_III_ decreased after surfactant indicating ventilation inhomogeneity improvement.

**Methods:**

Respiratory function traces of preterm infants resuscitated at birth were analysed. Ten capnograms were constructed for each infant: five pre- and post-surfactant. If a plateau was present S_III_ was calculated by regression analysis.

**Results:**

Thirty-six infants were included, median gestational age of 28.7 weeks and birth weight of 1055 g. Average time between pre- and post-surfactant was 3.2 min. Three hundred and sixty capnograms (180 pre and post) were evaluated. There was adequate slope in 134 (74.4%) capnograms pre and in 100 (55.6%) capnograms post-surfactant (*p* = 0.004). Normalised for tidal volume S_III_ pre-surfactant was 18.89 mmHg and post-surfactant was 24.86 mmHg (*p* = 0.006). An increase in S_III_ produced an up-slanting appearance to the plateau indicating regional obstruction.

**Conclusion:**

It was feasible to evaluate the alveolar plateau pre-surfactant in preterm infants. Ventilation inhomogeneity increased post-surfactant likely due to airway obstruction caused by liquid surfactant present in the airways.

**Impact:**

- Volumetric capnography can be used to assess homogeneity of ventilation by S_III_ analysis.
- Ventilation inhomogeneity increased immediately post-surfactant administration during the resuscitation of preterm infants, producing a characteristic up-slanting appearance to the alveolar plateau.
- The best determinant of alveolar plateau presence in preterm infants was the expired tidal volume.

## Introduction

Capnography allows for continuous measurement of carbon dioxide (CO_2_) expired from the large conducting and smaller gas exchanging airways during ventilation and can be represented relative to time or volume.^1^ The latter enables breath by breath analysis of carbon dioxide elimination in relation to tidal volume and by construction of a capnogram waveform can allow calculation of the end-tidal CO_2_ and physiological dead space.^2^ Furthermore, volumetric capnography can provide clinicians with valuable knowledge regarding the homogeneity of alveolar ventilation, adequacy of gas exchange and ventilation–perfusion (V/Q) relationships, provided the shape of the capnogram is well-defined.^3,4^ Three phases of expiration can be determined with the third (or alveolar) phase producing a plateau as pure alveolar gas is exhaled.^5^ The gradient of the alveolar plateau, S_III_, can be used as an index of alveolar ventilation inhomogeneity, with a steeper slope of the plateau phase indicating more severe ventilation inhomogeneity.^6^ Indeed, a strong correlation between V/Q ratio and S_III_ obtained from volumetric capnography has been demonstrated in an animal model of acute lung injury.^7^ Furthermore, the S_III_ results pre- and post-bronchodilator therapy in children with asthma showed a steeper alveolar plateau prior to treatment resulting from small airway obstruction.^8^

Spontaneously breathing infants with bronchopulmonary dysplasia have been shown to have an S_III_ steeper than their non-BPD preterm counterparts, suggestive of disease induced changes to the lung parenchyma adversely affecting V/Q matching.^9^ We have previously described a steeper S_III_ in invasively ventilated term or preterm infants who required higher levels of ventilatory support on the neonatal intensive care unit.^10^ The morphometric changes in alveoli and small airways as infants mature are thought to explain the mechanism behind the decrease in the slope of the alveolar plateau over time from infancy to adolescence, with one study demonstrating a smaller S_III_ as gestational age advances and tidal volumes increase.^11^ Constructing volumetric capnograms, however, can be methodologically difficult in the preterm population, in part due to their small tidal volumes, rapid breathing rates and shortened expiratory times.^12^

Exogenous surfactant is administered during the initial resuscitation of preterm infants at birth to prevent respiratory distress syndrome (RDS) and reduce ventilation–perfusion mismatch. Surfactant therapy can improve lung volume, oxygenation and atelectasis in newborn infants as demonstrated by a post-surfactant increase in functional residual capacity.^13^ Ventilation inhomogeneity can be assessed by moment analysis of the nitrogen washout curve according to an exponential lung model. Moment ratios, calculated from the percentage of total lung nitrogen volume and number of turnovers, are directly related to ventilation inhomogeneity. In a study of ventilated newborn infants (median gestational age 30 weeks) with RDS, homogeneity of ventilation assessed by the above techniques was determined pre- and post-surfactant. Post surfactant there was a significant drop in moment ratio (*p* < 0.05), with a single-compartment lung model fitting the washout data better post surfactant suggestive of improved homogeneity of ventilation (*p* < 0.01).^14^

Our aims were to describe the feasibility and methodology of analysing the alveolar plateau (S_III_) in preterm infants during resuscitation and to report on the changes in ventilation inhomogeneity pre- and post-surfactant administration.

## Methods

### Subjects

This was a retrospective analysis of respiratory function traces collected from a previous randomised controlled trial involving resuscitation of preterm infants (London Riverside NHS Research Ethics Committee: 16/LO/1718), with consent from the original study stating the data could be used to inform future research.

The traces of preterm infants less than 34 completed weeks of gestation were analysed if the infants required resuscitation with intubation and administration of endotracheal surfactant on the labour ward.

### Protocol

Infants were resuscitated using a continuous flow, pressure-limiting device (Neopuff infant resuscitator, Fisher and Paykel Healthcare, New Zealand). Management post-delivery was performed according to the Neonatal Life Support protocol (Resuscitation Council, UK) and the European Consensus Guidelines on RDS.^15^ Initial peak inspiratory pressures (PIP) of 20–25 cm H_2_O with a positive end-expiratory pressure (PEEP) of 4–5 cm H_2_O were used and increased according to clinician discretion if inadequate chest rise, no response to positive pressure ventilation or poor oxygenation. The fraction of inspired oxygen (FiO_2_) was initially set at 0.21 and was increased as necessary to maintain oxygen saturations between 92 and 96%. If infants were not responding to positive pressure ventilation via face mask they were intubated with shouldered Cole’s endotracheal tubes (ETTs) and given surfactant. The surfactant liquid preparation (Curosurf, Chiesi Italy) was administered via the ETT at a dose of 200 mg/kg.

### Respiratory function monitoring

Respiratory function monitoring was recorded via an NM3 respiratory profile monitor (Philips, Respironics) with customised Spectra software (Grove Medical, UK). The NM3 monitor has a combined flow, pressure, and mainstream carbon dioxide (CO_2_) sensor (Capnostat-5) with a dead space of 0.8 ml and a CO_2_ response time of less than 60 ms according to the manufacturer’s specifications. The NM3 Capnostat-5 device was placed into the ventilatory circuit between the Neopuff device and the ETT. During resuscitation the respiratory monitor displayed expired tidal volume, end-tidal CO_2_, peak inspiratory pressure and PEEP. Respiratory function monitoring traces analysed were those following intubation immediately pre- and post-surfactant administration once the ETT was reconnected to the Neopuff, prior to transferring the infant to the Neonatal Intensive Care Unit.

### Volumetric capnography indices

Data were extracted from the Spectra software into an Excel file with a sampling rate of 100 Hz. Ten volumetric capnograms were constructed for each infant: five pre and five post endotracheal surfactant administration. The flow signal was integrated over time to calculate the expired tidal volume. The anatomical dead space (Vd_ANA_) was calculated using the modified Bohr Enghoff (BE) equation (12): Vd_ANA_ (ml) = Vte * (PEtCO_2_ − PemeanCO_2_)/PEtCO_2_, where Vte is the expired tidal volume (ml), PEtCO_2_ is the end-tidal CO_2_ (mmHg) and PemeanCO_2_ is the volume average mean expired CO_2_ (mmHg). The mean expired CO_2_ is calculated as an integrated function of CO_2_ from the end of inspiration to the end of expiration. Partial pressure concentrations of expired carbon dioxide were reported rather than fractional concentrations due to the methodological feasibility of the former, in that exhaled CO_2_ as detected by our NM3 monitor was reported in mmHg. The expiratory time (ms) was taken from the start of negative flow to where the flow equals zero. The value of anatomical dead space calculated also includes the contribution from the apparatus dead space, which includes the ETT and NM3 Capnostat-5. The BE equation method was used preferentially over the method described by Fletcher using Fowler’s equal triangles as the BE method has been shown to be feasible for dead space measurements regardless of both the shape of the volumetric capnogram and the presence or absence of an alveolar plateau.^16,17^

**Fig. 1:**
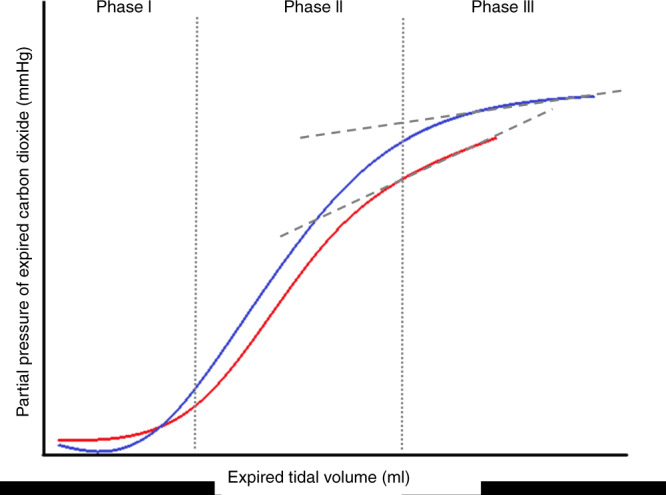
Volumetric capnogram from the same infant pre-surfactant (blue line) and post-surfactant (red line). The three phases are depicted and a regression line is fitted to the gradient of phase III.

The volumetric capnogram was split into three phases: phase I, phase II and phase III (Fig. 1).^5^ Phase I represents the first part of expiration whereby gas was expired from the large conducting, non-gas exchanging airways. Phase II corresponds to the mid-part of expiration and contains a mixture of gas from the large airways with alveolar gas from distal airways. Phase III describes the final phase of expiration with pure alveolar gas being expired with a resulting alveolar plateau. The gradient (slope) of the phase III part of the capnogram is called S_III_. The shape of each capnogram was evaluated and if an alveolar plateau was present S_III_ was calculated by fitting a regression line to this final phase, which relied on dynamic measurements taking each individual capnogram shape into account rather than calculating measurements at a fixed portion of the expired tidal volume, with a minimum of ten sample points required for S_III._^18,19^ The average values obtained from each of the five capnograms pre- and post-surfactant were calculated for each infant. Values of S_III_ were normalised to tidal volume to account for anthropometric differences.^10,11^ Furthermore, we present S_III_ values normalised to PemeanCO_2_ (ml^−1^) to account for differences in expired CO_2_. Feasibility of S_III_ analysis for each volumetric capnogram was assessed individually if an alveolar plateau was present allowing for S_III_ calculation. The feasibility was expressed as a percentage out of total number of capnograms analysed (Fig. 1). All evaluations were made by the same investigator who was blinded to the infant demographics at the time of analysis.

### Sample size

In a previous study the mean difference in normalised S_III_ between preterm infants who developed BPD and those who did not matched for a postmenstrual age of 44 weeks at study was 10.6 l^−^^1^ with a standard deviation of 14.4.^9^ To detect a significant difference of 10.6 in the S_III_ of infants pre- and post-surfactant with 85% power at the 5% significance level a sample size of 35 infants was required.

### Statistical analyses

The data were tested for normality using the Shapiro Wilk test and found to be non-normally distributed. Nonparametric tests were used with the Wilcoxon-matched pairs test to determine the change in capnography indices pre- and post-surfactant administration. The feasibility of S_III_ analysis was calculated as the percentage of the total that demonstrated presence of an alveolar plateau and allowed for S_III_ calculation. Differences between those capnograms that demonstrated an alveolar plateau and those that did not were assessed for statistical significance using the Mann–Whitney *U* test. The factors that were significantly different (*p* < 0.05) were inserted into a binary logistic regression model with the presence of alveolar plateau as the outcome variable. The performance of the characteristics that were significant in determining the presence of alveolar plateau was assessed by receiver operator characteristic (ROC) curve analysis and estimation of the corresponding area under the curve (AUC). Statistical analysis was performed using SPSS software version 26.0 (SPSS, New York).

## Results

Thirty-six infants were studied. They had a median (interquartile range, IQR) gestational age (GA) at birth of 28.7 (26.1–30.8) weeks, a birth weight of 1055 (811–1357) g and a birth weight *z*-score of −0.49 (−1.12 to 0.03) (Table 1). Eighteen infants (50.0%) were male. Fifteen infants (41.7%) were born by vaginal delivery and 21 infants (58.3%) by caesarean section. Thirty-one infants (86.1%) were exposed to antenatal corticosteroids. Three infants (8.3%) died prior to discharge.

**Table 1:** Demographic data of the preterm infants.

Gestational age (weeks)	28.7 (26.1–30.8)
Birth weight (kg)	1.06 (0.81–1.36)
Birth weight z-score	−0.49 (−1.12 to 0.03)
Male sex	18 (50)
Vaginal delivery	15 (41.7)
Antenatal corticosteroids	31 (86.1)
Ten-minute Apgar	9 (8–10)
Survival to discharge	33 (91.7)

Data are presented as median (interquartile range) or *N* (%).

The median time between pre- and post-surfactant analysis was 3.2 (1.7–4.8) minutes. The median end-tidal CO_2_ level pre-surfactant was 43.2 (29.6–51.7) mmHg and 33.1 (27.0–44.0) mmHg post-surfactant (*p* = 0.044). The median expired tidal volume decreased immediately post-surfactant administration compared to pre-surfactant (6.6 versus 9.3 ml/kg; *p* < 0.001). The median anatomical dead space to tidal volume ratio (Vd/Vt) was higher in infants post-surfactant compared to pre-surfactant (0.58 versus 0.54; *p* = 0.012). There was no significant difference in the maximal peak inspiratory pressure (PIP) used pre- and post-surfactant (*p* = 0.383) (Table 2).

**Table 2:** Respiratory function parameters of the preterm infants pre- and post-surfactant administration.

	Pre-surfactant	Post-surfactant	*p* value
End-tidal CO_2_ (mmHg)	43.2 (29.6–51.7)	33.1 (27.0–44.0)	0.044
Expired tidal volume (ml/kg)	9.4 (7.5–13.4)	6.3 (4.3–10.1)	<0.001
Anatomical dead space (ml/kg)	5.2 (4.1–6.4)	4.1 (2.7–5.5)	0.001
Maximal PIP (cm H_2_O)	26.9 (24.7–27.9)	26.6 (23.2–28.1)	0.383
Presence of alveolar plateau (%)	134 (74.4)	100 (55.6)	0.004
Non-normalised S_III_ (mmHg/ml)	1.58 (0.76–3.09)	2.44 (1.22–5.23)	0.005
Normalised S_III_ for tidal volume (mmHg)	18.89 (9.32–29.08)	24.86 (16.04–31.18)	0.006
Normalised S_III_ for PemeanCO_2_ (ml^−1^)	0.08 (0.03–0.20)	0.16 (0.06–0.43)	0.003
Expiratory time (ms)	572 (437–675)	499 (410–591)	0.051

Data are presented as median (interquartile range) or *N* (%).

Three hundred and sixty volumetric capnograms were evaluated for adequate slope definition (180 pre- and 180 post-surfactant). The alveolar plateau was present and analysable in 134 (74.4%) capnograms pre-surfactant and in 100 (55.6%) capnograms immediately post-surfactant (*p* = 0.004). The median (IQR) S_III_ pre-surfactant was 1.58 (0.76–3.09) mmHg/ml and 2.44 (1.22–5.23) mmHg/ml post-surfactant (*p* = 0.005). The median (IQR) normalised for tidal volume S_III_ pre-surfactant was 18.89 (9.32–29.08) mmHg and post-surfactant was 24.86 (16.04–31.18) mmHg (*p* = 0.006). The median (IQR) normalised for PemeanCO_2_ S_III_ pre-surfactant was 0.08 (0.03–0.20) ml^−1^ and post-surfactant was 0.16 (0.06–0.43) ml^−1^ (*p* = 0.003). The expiratory time of capnograms with an alveolar plateau was 572 (437–675) ms pre-surfactant compared to 499 (410–591) ms post-surfactant (*p* = 0.051).

Of the 180 volumetric capnograms that were constructed pre-surfactant administration the alveolar plateau was present in more mature [median GA 28.9 (27.0–30.1) weeks] compared to less mature infants [median GA 27.9 (24.9–30.1) weeks; *p* = 0.013]. There was no significant difference in birth weight where an alveolar plateau was present [1.1 (0.84–1.36) kg] compared to where it was absent [0.87 (0.66–1.29) kg; *p* = 0.071). The median expiratory time in all 180 capnograms was 540 ms, with the expiratory time significantly longer in those capnograms demonstrating presence of an alveolar plateau [580 (450–730) ms] compared to those without [390 (295–550) ms; *p* < 0.001]. Expired tidal volumes were also greater if an alveolar plateau was present [10.6 (8.4–16.8) ml/kg] compared to when it was absent [5.3 (4.3–6.7) ml/kg; *p* < 0.001). The correlation between expiratory time and expired tidal volume was moderate (*r* = 0.48; *p* < 0.001). Binary logistic regression analysis with gestational age, expiratory time and expired tidal volume as covariates showed that all three covariates remained independently associated with the presence of an alveolar plateau (Table 3). ROC analysis demonstrated an AUC of 0.92 for expired tidal volume in determining the presence of an alveolar plateau. A tidal volume greater than or equal to 7.0 ml/kg had 92% sensitivity and 82% specificity in discriminating whether a capnogram had an alveolar plateau (Table 4).

**Table 3:** Binary regression analysis with differences in characteristics between infants exhibiting a pre-surfactant alveolar plateau compared to those without.

	Alveolar plateau	No alveolar plateau	Adjusted *p* value
Gestational age (weeks)	28.9 (27.0–30.1)	27.9 (24.9–30.1)	<0.001
Expiratory time (ms)	580 (450–730)	390 (295–550)	0.043
Expiratory tidal volume (ml/kg)	10.6 (8.4–16.8)	5.3 (4.3–6.7)	<0.001

Data are presented as median (interquartile range).

**Table 4:** Areas under the receiver operator characteristic curves related to determination of presence of alveolar plateau.

	AUC	95% CI
Gestational age (weeks)	0.62	0.52–0.73
Expiratory time (ms)	0.75	0.66–0.84
Expiratory tidal volume (ml/kg)	0.92	0.87–0.97

## Discussion

We have demonstrated the feasibility of using volumetric capnography during resuscitation of preterm infants and that the presence of an alveolar plateau is best determined by a larger expiratory tidal volume. Furthermore, we have described an increase in ventilation inhomogeneity immediately post-surfactant therapy.

The increase in S_III_ and decrease in expired tidal volume that occurs immediately post-surfactant may be explained by the liquid surfactant preparation obstructing the small airways. Indeed complete airway obstruction post-surfactant administration in the delivery room has previously been described in a cohort of 16 infants, with similar changes in expired tidal volume to our current findings.^20^ The immediate increase in ventilation inhomogeneity could explain why some infants exhibit an acute deterioration following surfactant administration.^21^ The gradient of alveolar plateau post-surfactant demonstrated a ‘shark’s fin’ appearance to the shape of the capnogram similar to that observed in asthmatic patients presenting with small airway inflammation and obstruction prior to bronchodilator therapy.^22,23^

The feasibility in the current study of determining an alveolar plateau pre-surfactant was 74%; however, post-surfactant dropped to 56% secondary to the above-described obstructive findings. Previous descriptions of volumetric capnography derived calculations of dead space and S_III_ have been evaluated in one newborn animal model of ventilated surfactant-depleted lungs secondary to bronchoalveolar lavage (BAL). The number of capnograms without alveolar plateaus was significantly decreased following BAL, with no plateau phase in over three quarters of those with expiratory times of less than 200 ms, and with a feasibility rate of 50% in surfactant-depleted lungs increasing to as high as 90% in healthy lungs. The average expiratory time in our cohort pre-surfactant was longer at 540 ms and may explain our higher percentage of feasibility.^17^

Expiratory tidal volume and expiratory time were better determinants of alveolar plateau presence in our study than birth weight. Indeed a study performed in spontaneously breathing infants has described how capnography wave patterns are not affected by body weight per se but rather by maturity.^24^ By normalising the S_III_ to tidal volume we allowed ventilation inhomogeneity to be interrogated independently of significant changes in slope with expired tidal volume. That the best determinant of a presence of an alveolar plateau was the expiratory tidal volume, was an unexpected finding. A computer-simulated model has reported changes in ventilation inhomogeneity calculated by lung clearance index to be influenced by lung volume and dead space to tidal volume ratio, with reduced indices often misinterpreted as more homogeneous distribution of ventilation, suggesting ventilation homogeneity indices need to be considered in the context of tidal volumes and peak inspiratory pressures.^25^ We suggest the change in expiratory tidal volume and anatomical dead space to tidal volume ratio pre- and post-treatment in the current study was due to the surfactant preparation partially blocking exhalation of expiratory gas and not due to changes in ventilatory pressures influencing tidal volume delivery. It needs to be highlighted that values of S_III_ should be interpreted with caution when significant changes in tidal volume occur. The presence of an alveolar plateau could indeed indicate that delivery of tidal volume to the alveoli is large enough to overcome the dead space and allow for adequate alveolar ventilation and clearance of carbon dioxide.

The values of normalised for tidal volume S_III_ pre-surfactant (median 18.9 mmHg) in our study are higher than reported in the literature. Using the same method of analysis, we have previously demonstrated normalised for tidal volume values of S_III_ pre-extubation of 16.5 mmHg in preterm and 13.5 mmHg in term infants, measured at 30 and 40 weeks corrected gestational age, respectively.^10^ The higher values in the current study of infants, measured at a less mature gestational age of 28 weeks, may relate to the morphometric changes to alveolar lung growth that occur during maturation with a corresponding decrease in the slope of phase III.^11^ We further speculate that atelectasis and greater V/Q mismatch immediately post birth, prior to surfactant, might explain the larger values of S_III_, with improvement in respiratory disease and hence lower S_III_ in those infants studied at a later chronological time point. Contrary to this potential explanation however is a study of preterm infants with bronchopulmonary dysplasia measured at 44 weeks corrected gestational age, in which such infants had normalised for fraction of expired CO_2_ S_III_ values of 26.8 l^−1^ compared to term controls of 14.8 l^−1^. Suggesting that after the initial convalescent phase of disease, evolution of parenchymal lung changes with progressive V/Q mismatch may indeed increase indices of ventilation inhomogeneity.^9^ That study, however, was performed in spontaneously breathing infants and as such employed different equipment (face mask) and methodology of S_III_ calculation, hence, may not be fully comparable to our study. Furthermore, we acknowledge comparison of S_III_ between different studies may be difficult to evaluate given different rates of exhaled carbon dioxide; we have, therefore, presented S_III_ normalised to PemeanCO_2_ to account for this.

Our study has strengths and some limitations. This is the first study to evaluate the shape of the volumetric capnogram and to describe S_III_ during the resuscitation immediately post birth in preterm infants. Despite often a lack of alveolar plateau in critically ill neonates, attributable to small tidal volumes and V/Q mismatch in respiratory disease, even in plateau absence some clinical information from the capnogram can still be useful with end-tidal carbon dioxide being used to non-invasively estimate arterial carbon dioxide.^26^ Our findings may be used to inform clinicians regarding the effect of surfactant on ventilatory parameters immediately post administration; indeed, slower administration may be required to avoid partial airway obstruction. Volumetric capnography may further be useful in adjustment of ventilatory parameters post-surfactant administration until ventilation inhomogeneity improves.

Advances in technology have enabled development of mainstream and sidestream capnography devices with low apparatus dead space (<0.8 ml), low sampling rates and fast response times (<60 ms), making them more suited to the neonatal population;^12,27,28^ indeed, we used such a mainstream device in our current study. The use of Cole’s shouldered ETTs in minimising leak,^29^ together with the low dead space measuring apparatus^30^ allowed for calculation and construction of volumetric capnograms which was crucial to the methodology employed. The steeper alveolar plateau and increase in S_III_ secondary to small airway obstruction inhibited accurate interpretation of the capnogram shape in some infants post-surfactant and we were therefore only able to comment on the feasibility of S_III_ analysis in pre-surfactant volumetric capnograms reducing the numbers for analysis; however, 36 infants and 180 capnograms met our sample size calculation. Determining the PEtCO_2_ value when the alveolar plateau is poorly defined and short may not be representative of the alveolar CO_2_, we, therefore, determined the PEtCO_2_ at the end of expiration defined by the end of expiratory flow. Values of arterial carbon dioxide are not routinely collected on the labour ward and so we could not comment on the alveolar dead space which may influence ventilation inhomogeneity. In choosing not to analyse the data by automation using a fixed portion of phase III, our results may be affected by subjectivity; however, this study was the first to describe alveolar plateau analysis during resuscitation of premature infants. Future studies could incorporate automated analysis which would improve the potential for real time utilisation.

In conclusion, evaluation of the alveolar plateau was feasible in pre-surfactant volumetric capnograms and expired tidal volume was the major determinant of an alveolar plateau presence. Ventilation inhomogeneity as assessed by S_III_ increased immediately post-surfactant administration likely due to airway obstruction caused by the liquid surfactant in the small airways.
